# Supportive supervision and constructive relationships with healthcare workers support CHW performance: Use of a qualitative framework to evaluate CHW programming in Uganda

**DOI:** 10.1186/s12960-018-0272-1

**Published:** 2018-02-13

**Authors:** Teralynn Ludwick, Eleanor Turyakira, Teddy Kyomuhangi, Kimberly Manalili, Sheila Robinson, Jennifer L. Brenner

**Affiliations:** 1 School of Public Health, University of Alberta, 116 St & 85 Ave, Edmonton, AB T6G 2R3 Canada; 2 Department of Community Health, Faculty of Medicine, Mbarara University of Science and Technology, P.O BOX 1410 Mbarara, Uganda; 3 Maternal, Newborn, and Child Health Institute, Mbarara University of Science and Technology, P.O BOX 1410 Mbarara, Uganda; 4 Department of Community Health Sciences, Cumming School of Medicine, University of Calgary, 2500 University Dr NW, Calgary, AB T2N 1N4 Canada; 5 Pediatrics, Cumming School of Medicine, University of Calgary, 2500 University Dr NW, Calgary, AB T2N 1N4 Canada

## Abstract

**Background:**

While evidence supports community health worker (CHW) capacity to improve maternal and newborn health in less-resourced countries, key implementation gaps remain. Tools for assessing CHW performance and evidence on what programmatic components affect performance are lacking. This study developed and tested a qualitative evaluative framework and tool to assess CHW team performance in a district program in rural Uganda.

**Methods:**

A new assessment framework was developed to collect and analyze qualitative evidence based on CHW perspectives on seven program components associated with effectiveness (selection; training; community embeddedness; peer support; supportive supervision; relationship with other healthcare workers; retention and incentive structures). Focus groups were conducted with four high/medium-performing CHW teams and four low-performing CHW teams selected through random, stratified sampling. Content analysis involved organizing focus group transcripts according to the seven program effectiveness components, and assigning scores to each component per focus group.

**Results:**

Four components, ‘supportive supervision’, ‘good relationships with other healthcare workers’, ‘peer support’, and ‘retention and incentive structures’ received the lowest overall scores. Variances in scores between ‘high’/‘medium’- and ‘low’-performing CHW teams were largest for ‘supportive supervision’ and ‘good relationships with other healthcare workers.’ Our analysis suggests that in the Bushenyi intervention context, CHW team performance is highly correlated with the quality of supervision and relationships with other healthcare workers. CHWs identified key performance-related issues of absentee supervisors, referral system challenges, and lack of engagement/respect by health workers. Other less-correlated program components warrant further study and may have been impacted by relatively consistent program implementation within our limited study area.

**Conclusions:**

Applying process-oriented measurement tools are needed to better understand CHW performance-related factors and build a supportive environment for CHW program effectiveness and sustainability. Findings from a qualitative, multi-component tool developed and applied in this study suggest that factors related to (1) supportive supervision and (2) relationships with other healthcare workers may be strongly associated with variances in performance outcomes within a program. Careful consideration of supervisory structure and health worker orientation during program implementation are among strategies proposed to increase CHW performance.

## Background

Globally, community health worker (CHW) programs have been positioned as an important strategy for achieving the Millennium Development Goals and now for progressing towards the universal health coverage targets set out in the 2030 Agenda and Sustainable Development Goals [1–3]. CHWs are generally understood to be a cadre of lay health workers, typically selected by and from their own communities, who receive short training on a range of health education, health promotion activities, and/or basic treatment related to the intervention, but do not hold a formal professional certification [4, 5]. Evidence demonstrates that CHWs can have a significant impact on health outcomes, including on maternal, newborn, and child health in low- and middle-income country settings [5–9]. Yet despite growing global experience with CHWs, key program implementation challenges remain, creating barriers to scale-up and sustainability for CHW programs [10–12].

Health intervention research has traditionally focused on *whether* an intervention works and not *why* and under *what conditions* it is effective [13, 14]. With regard to CHW programs, there is a lack of evidence on what operational factors affect CHW performance, and a corresponding lack of tools to undertake this type of assessment [15–17]. Furthermore, insufficient space is given to CHW voices as part of evidence-gathering and assessment processes [8]. Recognizing this gap, there is a need to better identify which system- and program-level processes, structures, and strategies contribute to the implementation of effective interventions [7, 18–20]. To better understand the workings of CHW interventions and address implementation challenges, greater attention needs to be placed on developing and applying innovative and rigorous systems-oriented methods that can provide evidence on how different elements of the system are functioning, how they are interacting, and their impact on program outcomes [13, 19].

This paper describes a study which was embedded within a larger, end-of-project, qualitative evaluation of a scaled, 3-year, district-level, maternal, newborn, and child health (MNCH) intervention. The project included CHW selection, training and support within a full district in rural, southwest Uganda [21]. A conceptual framework for CHW effectiveness was developed and applied in this study to test its usage in comparing the performance of CHW teams and generating evidence on factors that contribute to variances in CHW team performance.

### Study setting

Healthy Child Uganda (HCU) is a Ugandan-Canadian university partnership which has implemented CHW and maternal, newborn, and child health programming since 2003. Together, Mbarara University of Science and Technology (Uganda), the University of Calgary (Canada) and the Canadian Paediatric Society promote health system strengthening in rural districts using MNCH as an entry point. Through research, HCU has sought to better understand factors related to effective implementation and sustainability of related programming [22–24].

CHWs (known as village health teams in Uganda) are community-based volunteers who encourage community participation in health, link communities to the formal health service delivery system, and help bridge the current health human resource gap especially in rural or peripheral areas [25]. Responsibility for implementation of village health teams is decentralized to the Districts [21]. A key component of the HCU-supported intervention was operationalizing volunteer CHWs to act in a health promotion role with a focus on MNCH.

Between 2012 and 2014, an intervention was implemented by HCU to scale up MNCH programming throughout Bushenyi district. Bushenyi is located in southwest Uganda with a population of about 234,000 [26]. Access to quality health services is limited and health indicators in the district are poor, with under 5 child mortality estimated at 54.5/1000 (based on HCU baseline survey data conducted in 2012). A comprehensive evaluation of the intervention was undertaken which included pre/post intervention MNCH outcome data, and a qualitative study to better understand progress and processes to refine the model and recommend best practices.

Funded by the Canadian Department of Foreign Affairs Trade and Development, through the Muskoka Initiative (Project S065346-001), the project supported capacity-development at three levels: (1) district health system (information systems; transport/referral protocols; planning and leadership); (2) health facilities (equipment/facility upgrades, MNCH clinical training; and (3) CHW scale up (training CHWs and CHW supervisors; collection and integration of community-level health data). Implementation of field activities was led by the district using the ‘MamaToto Approach’ developed by HCU [27].

### Intervention

During the intervention over 1600 CHWS were trained (average 3; SD 1.0 per village). CHWs from villages within the same administrative unit known as a ‘parish’ were grouped together, resulting in teams of about 20–30 CHWs (average 26.7; SD 13.1). Training included 5 days of initial training (mandatory government standard) with five supplementary days covering MNCH and nutrition topics. CHW supervisors were appointed by the District Health Team and attended 2 weeks of training on parallel topics to those received by CHWs. CHWs were expected to support MNCH promotion activities (i.e., health talks; home visits; early assessment; treatment; referral for sick children and pregnant women; child/pregnancy registration) and encourage community initiatives (i.e., healthy home competitions; demonstration gardens; community action plans). CHWs from each parish team met initially on a monthly then quarterly basis and were supervised by a local health worker at a nearby health center. CHWs were volunteers and not remunerated; incentives included t-shirts, training manuals, training allowances (~$1 per day), and educational materials as job aids.

## Methods

### Study design

While the literature identifies various factors related to CHW performance, the relative impact of these factors on performance is not clear? What elements of the CHW system (when functioning well or poorly) have the greatest impact on performance? A conceptual framework for CHW effectiveness and rating tool was developed and applied in this study to test its usage in comparing the performance of CHW teams and generating evidence on factors that contribute to variances in CHW team performance. The study design entailed the following six steps (described below).

#### Conceptual framework

**Fig. 1:**
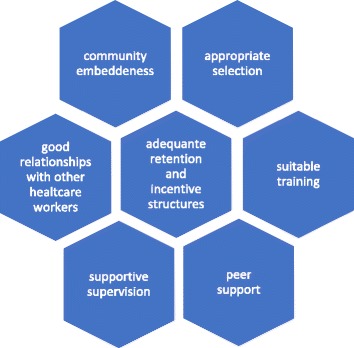
7-Component CHW Effectiveness Framework

A qualitative conceptual framework (Fig. 1) was developed to describe key components related to effective CHW programming: (1) appropriate selection, (2) suitable training, (3) community embeddedness, (4) peer support, (5) supportive supervision, (6) good relationships with other healthcare workers, (7) adequate retention and incentive structures. The framework integrates program components important for CHW effectiveness identified in a literature review by Campbell and Scott [28], the Community Owned Resource Person Model (CORP) developed by HCU [29] and practical input from the HCU field team. The framework includes program components about which CHWs and supervisors could be questioned. Definitions were established for each effectiveness component. This CHW effectiveness framework served as the basis for qualitative questioning during focus groups which probed into the functioning of each of the seven components.

#### Performance level ranking

Six field staff pre-ranked CHW teams by perceived performance levels based on field observation. The field staff were individuals who had provided external (i.e., outside-of-government health system) supportive supervision two to four times per year to CHW teams. Field staff were provided a list of all CHW teams and asked to assign each group a relative performance level (‘high’, ‘medium’ or ‘low’) compared to other CHW teams. No instruction was provided by the researchers to the field staff on how to determine the classification. Classification was determined by group consensus among the field staff and assisted by a set of guiding indicators developed by the group. Holding regular meetings, carrying out household house visits, relationships with health centers, and showing initiative though cooperative activities with other CHW teams, and managing income generating activities are examples of the guiding indicators used by the field staff. However, there were no standardized benchmarks for each performance level. The result was 25 teams (39%) identified as ‘high’-performing, 28 (44%) as ‘medium’-performing, and 11 (17%) as ‘low’-performing. This initial categorization was conducted in order to facilitate comparison of experiences between stronger and weaker teams, and identify factors that may be contributing to variances in performance.

#### Recruitment and sampling

All 64 CHW teams in the intervention were trained in MNCH. Two CHW teams from each performance level (‘high’, ‘medium’, ‘low’) were randomly sampled for participation in focus group discussions (FGDs). A purposeful, stratified sampling approach combined ‘typical case’ sampling (average cases) with intensity sampling (strong and weak cases) to capture the variation of experiences from ‘low’-, ‘medium’-, and ‘high’-performing teams [30]. Following completion of the initial six FGDs, two additional low-performing CHW teams were randomly selected. This second set of FGDs was conducted to better understand thematic differences that facilitators identified in FGDs with low-performing teams compared to ‘medium’- and ‘high’-performing teams. As CHW teams are comprised of up to 30 individuals, project registers were used to randomly select 12 members from each team to attend the FGD in their area. Non-attendees were not replaced.

#### Data collection and management

Focus group guides were developed de novo and used a semi-structured ‘questioning route’ [31] to examine each of the seven components identified in the framework. Focus group guides were translated into the local dialect (Runyankole), back translated and piloted for accuracy. The FGDs were facilitated by locally hired and trained research assistants who were familiar with the context and language and did not have a role in implementing CHW activities. Participant consent was obtained. FGDs were recorded, translated, and transcribed into English for analysis.

#### Analysis

Directed content analysis [32] was used to deductively code content from each FGD transcript to each of the seven components identified in the CHW effectiveness framework. NVivo 10 was used to assist in coding.

Based on the 7-Component CHW Effectiveness Framework, a Likert-style tool was developed de novo (by TL) to evaluate CHW team performance within each pre-identified component. Relative scores (1: poor performance; 2: mixed performance; 3: functional performance; 4: strong performance) were assigned based on the FGD responses recorded in the transcripts. A set of evaluative criteria was developed to support consistency in assigning scores for each component. An example of the scoring criteria for the component ‘Supportive Supervision’ is provided in Table 1.

**Table 1:** Sample of criteria for CHW effectiveness rating tool

Component	‘Poor’ performance	‘Mixed’ performance	‘Functional’ performance	‘Strong’ performance
Supportive supervision	Majority of CHW participants identify major issues with supervisor relationship.	CHW participants convey divided perception of supervisor relationship. More than a few issues identified.	Majority of CHW participants describe positive supervisor relationship; describe regular meetings, mentorship and respectful relationships. Few issues identified.	Almost all or all CHW participants describe strongly positive supervisor relationship; describe regular meetings, mentorship and respectful relationships; identify supervisor actions above and beyond expected (i.e., regular participation alongside CHWs during community education).

Using results from the content analysis, scoring of each of the seven effectiveness components per CHW team was performed (TL). A second analyst validated the scoring by reviewing a sub-set of the categorization performed by the primary analyst. Cumulative scores for each CHW team for each of the seven components were tallied in order to identify patterns in performance across and between teams.

## Results

Transcripts from eight FGDs (average of 10 participants per group; 79% female) were analyzed. Two FGDs were from ‘high’-performing teams, two from ‘medium’-performing teams and four from ‘low’-performing teams. There were about two ‘no-shows’ for each group. No refusals to participate were noted. Reasons for non-participation included sickness and attending funerals. Scores by ‘effectiveness component’ and by CHW team are shown in Table 2.

**Table 2:** Scoring of CHW effectiveness framework components

	Scores for ‘high’/‘medium’-performing teams				Scores for ‘low’-performing teams			
Effectiveness component	CHW team #1	CHW team #2	CHW team #3	CHW team #4	CHW team #5	CHW team #6	CHW team #7	CHW team #8
Supportive supervision	3	4	4	2	1	3	1	3
Appropriate selection	4	3	3	4	3	2	3	3
Suitable training	4	4	3	3	3	3	3	3
Adequate retention and incentive structures	3	3	4	3	2	1	3	3
Good relationships with other healthcare workers	2	2	3	4	1	1	1	2
Community embeddedness	3	4	4	3	3	3	2	3
Peer support	1	4	4	2	4	1	3	1
	Mean: 2.9	Mean: 3.4	Mean: 3.6	Mean: 3.0	Mean: 2.4	Mean: 2.0	Mean: 2.3	Mean: 2.6

1 = poor performance, 2 = mixed performance, 3 = functional performance, 4 = strong performance

Average scores for CHW teams ranged from 2.0 to 3.6 and from 2.0 to 3.3 across the ‘effectiveness components’ (where higher scores represent better performance). Scores assigned during the FGD analysis aligned with the field-staff performance rankings, in that the lowest four scores were all assigned to the teams ranked as ‘low’-performing by the field staff. ‘High’- and ‘medium’-performing teams received higher cumulative scores (total scores for all seven components) than did ‘low’-performing teams (mean 3.2 for ‘high’/‘medium’-performing; 2.3 ‘low’-performing). ‘High’- and ‘medium’-performing teams also received more ‘strong’ and ‘functional’ scores than low-performing teams, and were less likely to receive a ‘poor’ score for any of the seven components.

By component, the lowest numeric scores across all teams were seen for ‘good relationships with other healthcare workers’ (average 2.0) and ‘supportive supervision’ (average 2.6), while they were highest for ‘suitable training’ (average 3.3). Three components (‘suitable training’; ‘community embeddedness’; ‘appropriate selection’) received an average score greater than ‘3’ representing a *functional* score using this tool’s scale.

Certain components had a larger variance in scores when comparing ‘high’/‘medium’- versus ‘low’-performing teams (Table 3). Of the seven components, the variance in scores between ‘high’/‘medium’- and ‘low’-performing teams were greatest for the components related to ‘supportive supervision’, and ‘good relationships with other healthcare workers’. Components with the smallest variance in scores (least sensitive to variance) between ‘high’/‘medium’- and ‘low’-performing CHW teams were ‘suitable training’ and ‘peer support’. ‘Suitable training’ received both the highest average score, with all eight CHW teams receiving a score of at least 3, as well as the smallest variance in scores between ‘high’/‘medium’- and ‘low’-performing groups. In contrast, ‘good relationships with other healthcare workers’ received the lowest average score across all eight CHW teams and the highest variance in scores between ‘high’/‘medium’- and ‘low’-performing teams.

**Table 3:** Average scores by CHW effectiveness framework components

	Average score per component for all CHW teams sampled (*n* = 8)	Average score per component for ‘high’/‘medium’-performing CHW teams (*n* = 4)	Average score per component for ‘low’-performing CHW teams (*n* = 4)	Difference between average component score in ‘high’/‘medium’ vs ‘low’ teams
Supportive supervision	2.6	3.3	2.0	1.3
Appropriate selection	3.1	3.5	2.8	0.7
Suitable training	3.3	3.5	3.0	0.5
Adequate retention and incentive structures	2.8	3.3	2.3	1.0
Good relationships with other healthcare workers	2.0	2.8	1.3	1.5
Community embeddedness	3.1	3.5	2.8	0.7
Peer support	2.5	2.8	2.3	0.5

### Analysis of ‘supportive supervision’ and ‘good relationships with other healthcare workers’

While thematic analysis was conducted for all seven components, the following section focuses on the two components—‘supportive supervision’ and ‘good relationships with other healthcare workers’—which had the largest variance in scores between ‘high’/‘medium’- and ‘low’-performing teams; these components also received low overall scores. The level of ‘supportive supervision’ relates to the direct relationship with the individual who has primary responsibility for oversight of CHWs. ‘Good relationship with other healthcare workers’ relates to the level of respect and quality of interaction between CHWs and other health professionals in the healthcare system, including physicians, nurses, and administrators. While these two components may be linked, they are distinct factors represented in the literature.

Two of the four low-performing teams reported significant gaps or no contact with supervisors. Lack of supervision was more common in cases when supervisors were based at the higher-level facility located in the main town, and with replacements of transferred supervisors. CHW associated positive relationships with supervisors with more frequent contact, opportunity for constructive feedback and advice, and demonstration of respect. Close relationships with supervisors were reported to be highly motivating for CHWs. Conversely, a lack of supervisory support was associated with lack of motivation. Contrasting experiences are described below:

*Our supervisor has been there since we started this program. Like sometime back when we had challenges, she would be the one calling CHWs directly... For everything we are to do, she is always leading us and steering everything. When you find her in the hospital even late, she attends to you. To me she has supported us and done almost everything for us [‘high’/‘medium’-performing CHW team member].*

*Sometimes we [CHWs] want to come up [do better] but people let us down. Like for example, we last met that health worker [supervisor] one year ago. We have only met her once ever since we started training. So we don’t see any teamwork with our supervisor [‘low’-performing CHW team member].*

Three of the four ‘high’- and ‘medium’-performing CHW teams provided evidence of involvement in health facility activities (e.g., assisting with immunization activities) while only one of the four low-performing CHW teams provided examples of facility engagement. Of the five CHW teams which voiced challenges with the referral system, four were ‘low’-performing. Working referral systems, in particular, were seen as an important sign of respect and validation of a CHW’s work, whereas CHWs felt that referrals that were rejected or ignored undermined their relationships with the community and the value of their work. Contrasting experiences are described below:

*When you have the phone number of the healthcare worker, you call her to let her know that you have referred a woman; when the woman reaches the clinic and delivers, the healthcare worker calls to tell you that the woman you sent has given birth. So that’s the relationship we have in the village and with the healthcare workers [‘high’/‘medium’-performing CHW team member].*

*The healthcare workers don’t support our work at all…they don’t respect us at all. They don’t think the referrals are important to them; they just throw them away and don’t even want to hear about them. They even ask patients whether they think it is the referral we give them that will treat them [‘low’-performing CHW team member].*

## Discussion

This study builds on the CHW effectiveness literature and existing frameworks [15, 33] by packaging specific program-level components into a single program evaluation tool. The CHW effectiveness literature has largely been quantified in terms of health outcomes; the paucity of CHW process studies is an identified gap [6, 18]. This implementation study emphasizes CHW process outcomes which may be closely linked to sustainability and program success and are important factors for scale-up and longer-term impact.

This particular study used a practical evidence- and experienced-based conceptual framework to evaluate and explain implementation strengths and weaknesses and their contribution to (varied) performance outcomes. Testing of this program evaluation tool suggest that CHW effectiveness components with large variance in scores between ‘high’/‘medium’- versus ‘low’-performing teams may be associated with performance outcomes. Our study found that components related to ‘supportive supervision’ and ‘good relationships with other healthcare workers’ were most important in differentiating performance levels of CHW teams. This study reinforces evidence that supervision and referral-liaison provided by other healthcare workers support are among the most important program elements affecting CHW effectiveness [19, 34].

We hypothesize that strong supervision helps foster a ‘virtuous’ cycle contributing to CHW confidence, cohesion, referral effectiveness, recognition in the community, and a sense of connectedness to the health system—factors linked to motivation. Conversely, weak or absent supervision seems to foster a ‘vicious’ cycle contributing to a feeling of neglect, lack of technical support and mentoring, weak referral systems, and poorer treatment by other healthcare workers—factors associated with lower motivation and performance. Such linkages may help explain why low-performing teams in our study received lower scores for components related to relationships with other healthcare worker and supportive supervision. Other authors similarly highlight the importance of regular supervisor interactions [35], the role of supervisors in reinforcing CHW legitimacy among community members [36], and building respect for CHWs by local health services [19, 37], and link these factors to CHW motivation and performance^.^ [38, 39].

To maximize CHW program effectiveness, policymakers and program implementers should consider strategies which orient healthcare workers, develop strong supervisory structures and budgetary support for supervisory activities, and provide channels for positive CHW healthcare worker interaction and communication. Exploration of innovative supervisory models, such as through group, community, and peer supervision could strengthen current healthcare worker-focused supervisory models as suggested by Lunsfeld [40] and Crigler [41].

Further development and testing of this innovative CHW program evaluation tool and similar tools could enhance more effective implementation as CHW programs are scaled up globally. Important limitations of our study include the use of relative scores, a lack of blinding to performance levels, and testing on a single program where a number of components had little variance in implementation. However, this tool accommodates heterogeneity in program design and generates contextually specific and locally relevant evidence based on universally applicable themes. The tool would be strengthened further by testing with a range of programs to determine generalizability of results, triangulating qualitative evidence-based scores with quantitative output data, and testing for inter-rater reliability. In the rural, southwestern Uganda study context, the small variance in components related to training, community embeddedness, and peer relationships was likely influenced by consistency in processes related to selection and training where clear guidelines and support for these processes were provided by the project. Gathering more nuanced perspectives on several of the CHW effectiveness components may help to further identify differentiating factors between higher- and lower-performing teams. For example, it would be useful to investigate relationships with local councilors as distinct from relationships with villagers, the role of peer leadership and group dynamics within CHW teams, and different facets of incentives and motivation. Such information may help to yield more precise information on performance levels.

## Conclusions

CHW programming has become a major strategy for task-shifting in resource-constrained contexts. Despite large funding investments, major implementation challenges remain. The lack of detailed, comparable, and systematic information on program design and implementation is hindering the global community’s capacity to effectively address these challenges and answer important questions about effective program design. For example, understanding what supervisory or incentive models work and under what conditions requires that the global health community develop innovative process-oriented, assessment tools to systematically evaluate and compare how various elements of program design affect overall program effectiveness. This study makes one of the first attempts to do so. Findings from the use of a qualitative, multi-component tool developed and applied in this study suggest that factors related to supportive supervision and relationships with other healthcare workers may be strongly associated with variances in performance outcomes within a program. Putting in place strategies and structures to support positive and mutually constructive engagements and relationships between CHWs, their supervisors, and the health system are proposed to support program effectiveness. With CHWs increasingly being turned to as the go-to community resource, understanding the enabling factors needed for a strong program will be essential for maintaining adequate CHW performance and retention.
